# Mrc1-Dependent Chromatin Compaction Represses DNA Double-Stranded Break Repair by Homologous Recombination Upon Replication Stress

**DOI:** 10.3389/fcell.2021.630777

**Published:** 2021-02-15

**Authors:** Poyuan Xing, Yang Dong, Jingyu Zhao, Zhou Zhou, Zhao Li, Yu Wang, Mengfei Li, Xinghua Zhang, Xuefeng Chen

**Affiliations:** Hubei Key Laboratory of Cell Homeostasis and the Institute for Advanced Studies, College of Life Sciences, Wuhan University, Wuhan, China

**Keywords:** replication checkpoint, Mrc1, DNA double-stranded breaks, homologous recombination, replication stress

## Abstract

The coordination of DNA replication and repair is critical for the maintenance of genome stability. It has been shown that the Mrc1-mediated S phase checkpoint inhibits DNA double-stranded break (DSB) repair through homologous recombination (HR). How the replication checkpoint inhibits HR remains only partially understood. Here we show that replication stress induces the suppression of both Sgs1/Dna2- and Exo1-mediated resection pathways in an Mrc1-dependent manner. As a result, the loading of the single-stranded DNA binding factor replication protein A (RPA) and Rad51 and DSB repair by HR were severely impaired under replication stress. Notably, the deletion of *MRC1* partially restored the recruitment of resection enzymes, DSB end resection, and the loading of RPA and Rad51. The role of Mrc1 in inhibiting DSB end resection is independent of Csm3, Tof1, or Ctf4. Mechanistically, we reveal that replication stress induces global chromatin compaction in a manner partially dependent on Mrc1, and this chromatin compaction limits the access of chromatin remodeling factors and HR proteins, leading to the suppression of HR. Our study reveals a critical role of the Mrc1-dependent chromatin structure change in coordinating DNA replication and recombination under replication stress.

## Introduction

Maintenance of genome stability relies on checkpoint signaling pathways that perceive DNA damage or replication stress to initiate a cellular response that coordinates DNA replication, repair with the cell cycle progression. During the S phase, cells are particularly vulnerable since the progression of replication forks can be impeded by numerous physical, chemical, or genetic perturbations, such as the hard-to-replicate regions, natural pausing sites, chromatin-bound proteins, secondary DNA structures, active transcription, or DNA replication inhibitors (Giannattasio and Branzei, 2017; Pardo et al., 2017). These barriers can cause the uncoupling of DNA helicase and replicative polymerases or between leading or lagging strand synthesis, leading to the accumulation of single-stranded DNA (ssDNA) (Garcia-Rodriguez et al., 2018). Replication protein A (RPA), the first responder of ssDNA, binds the exposed ssDNA, leading to the recruitment of the checkpoint kinase Mec1–Ddc2 complex and the activation of the S phase checkpoint (Zou and Elledge, 2003; Chen and Wold, 2014).

In yeast, the S phase checkpoint pathway is comprised of two branches: the DNA damage checkpoint and the DNA replication checkpoint (Pardo et al., 2017). Both pathways are initiated by the sensor kinase Mec1 and converge on the effector kinase Rad53 (Pardo et al., 2017). However, they differ in the mediator proteins. The DNA damage checkpoint relies on the adaptor protein Rad9, while the replication checkpoint depends on the replisome component Mrc1 (Claspin in human) (Alcasabas et al., 2001; Prado, 2014; Pardo et al., 2017). Upon replication stress, Mrc1 is phosphorylated by Mec1, and the modified Mrc1 interacts with the FHA domain of Rad53 to promote its activation (Tanaka and Russell, 2001; Smolka et al., 2006; Xu et al., 2006). Activation of the replication checkpoint turns on a cassette of events, leading to stabilization of stalled forks, suppression of late fired origins, induction of DNA damage response genes, upregulation of dNTP pools, and arrest of the cell cycle (Pardo et al., 2017).

Mrc1 is also a replisome component traveling with replication forks, and it interacts with DNA polymerase epsilon and Mcm6 (Katou et al., 2003; Lou et al., 2008; Komata et al., 2009). Mrc1 forms a complex with Csm3 and Tof1 at normal or stalled forks (Katou et al., 2003; Noguchi et al., 2004; Calzada et al., 2005). The complex fulfills a structural role required for normal replication fork progression in addition to its role in the S phase checkpoint activation (Calzada et al., 2005; Szyjka et al., 2005; Tourriere et al., 2005; Bando et al., 2009; Pardo et al., 2017). One of the essential functions of the replication checkpoint is to stabilize forks and preserve the ability of forks to synthesize DNA after exposure to genotoxic stress (Cortez, 2015). In the absence of a functional replication checkpoint, replication can be terminated irreversibly upon methyl methanesulfonate (MMS) or hydroxyurea (HU) treatment (Tercero and Diffley, 2001; Tercero et al., 2003; Lopes et al., 2006), leading to the accumulation of pathological structures at forks, such as ssDNA gaps and reversed, collapsed, or broken forks (Sogo et al., 2002; Cobb et al., 2003, 2005; Cortez, 2015; Rossi et al., 2015; Pardo et al., 2017). These structures can generate DNA double-stranded breaks (DSBs), a highly deleterious form of DNA lesion threatening genome stability. Homologous recombination (HR) is an essential pathway for the recovery of stalled or collapsed forks and the repair of DSBs (Kowalczykowski, 2015; Haber, 2016; Kramara et al., 2018).

HR utilizes a homologous template, usually a sister chromatid, to direct the repair and generally produces accurate repair products (Kowalczykowski, 2015; Haber, 2016). Deficiencies in HR cause genome instability and cancer (Prakash et al., 2015). During HR, the 5′-ends of DSBs are initially processed by the Mre11–Rad50–Xrs2 complex (MRE11-RAD50-NBS1 in mammals) in conjunction with Sae2 (CtIP in mammals) (Cannavo and Cejka, 2014). Further nucleolytic degradation of the 5′-ends is carried out by the exonuclease Exo1 or the Sgs1 helicase (BLM or WRN in mammals) and Dna2 nuclease (Mimitou and Symington, 2008; Zhu et al., 2008; Cejka et al., 2010; Niu et al., 2010). The 3′-tail ssDNA revealed by resection recruits the conserved ssDNA binding factor RPA. The recombinase Rad51 subsequently replaces RPA on ssDNA with the assistance of the mediator protein Rad52 (BRCA2 in mammals). This leads to the formation of Rad51–ssDNA nucleofilament that performs homology search and strand invasion of homologous duplex DNA (Kowalczykowski, 2015). The DNA synthesis primed by the 3′-end of the invading strand extends D-loop to complete the repair (Kowalczykowski, 2015).

In support of the role of HR in the recovery of stalled forks or in the repair of broken forks, cells deficient in members of the Rad52 epistasis group are sensitive to replication inhibitors (Chang et al., 2002; Lundin et al., 2005). However, paradoxically, multiple evidence showed that the replication checkpoint plays a role in inhibiting HR repair during the S phase. First, Mec1 prevents the formation of Rad52 foci upon HU or MMS treatment in the S phase in an Mrc1-dependent manner (Lisby et al., 2004; Alabert et al., 2009; Gonzalez-Prieto et al., 2013). Second, DSB end resection and HR repair were inhibited when the replication checkpoint is activated by HU or MMS treatment (Alabert et al., 2009; Barlow and Rothstein, 2009). The resection enzyme Exo1 is also negatively regulated by Rad53 to suppress its activity (Cotta-Ramusino et al., 2005; Smolka et al., 2007; Morin et al., 2008). To reconcile these conflicting observations, Alabert et al. (2009) proposed that the DNA replication checkpoint differentially regulates recombination at DSBs and stalled forks (Alabert et al., 2009; Prado, 2014). This differential regulation on HR repair appears to be critical for preserving the integrity of stalled forks (Pardo et al., 2017). However, a critical question that remains unclear is how HR repair is suppressed by replication stress.

In this study, we showed that the Mrc1-dependent replication stress signaling plays a critical role in repressing the 5′-end resection of the HO endonuclease-induced DSB and HR repair. We found that both Sgs1/Dna2 and Exo1 resection pathways are suppressed, and this suppression is relieved by the deletion of *MRC1*, but not *RAD9*. The suppression of resection is specifically mediated by Mrc1 since it is independent of Csm3, Tof1, or Ctf4. Finally, we showed that replication stress induces global chromatin compaction, which blocks the recruitment of the chromatin remodeling factors Ino80, RSC, and Fun30 that are known to function in resection. Our studies provide insight into how HR repair is repressed by replication stress and reveal a critical role of Mrc1-mediated chromatin compaction in coordinating replication and recombination.

## Materials and Methods

### Yeast Strains

The yeast strains used in this study are derivatives of JKM139 (*ho MAT****a*** *hml::ADE1 hmr::ADE1 ade1-100 leu2-3,112 trp1::hisG' lys5 ura3-52 ade3::GAL::HO*) or tGI354 (*MATa-inc arg5,6::MATa-HPH ade3::GAL::HO hmr::ADE1 hml::ADE1 ura3-52*). The genotypes for these strains are listed in Supplementary Table 1. The yeast strains were constructed with standard genetic manipulation.

### Analysis of 5′-End Resection by Southern Blot

Yeast cells were grown overnight in the pre-induction YEP–raffinose medium (1% yeast extract, 2% peptone, and 2% raffinose) to a density of ~1 × 10^7^ cells/ml. DSB induction was initiated by adding 2% galactose. For testing the resection under replication stress, 200 mM HU (final concentration) was added to each sample when starting the galactose induction. Samples were collected at different time points following break induction. DNA isolated by glass bead disruption using a standard phenol extraction method was digested with *E*coRI and separated on 0.8% agarose gels. The resolved DNA was transferred onto a Nylon hybridization transfer membrane (Perkin Elmer). Radiolabeling of DNA probes was carried out according to the manufacturer's instructions (Takara). Southern blotting and hybridization were carried out as described previously (Chen et al., 2012). The signal on the phosphor screen was captured by scanning with an OptiQuant Cyclone Plus machine (Perkin Elmer). Quantities of DNA loaded on gels for each time point were normalized using the *TRA1* DNA probe. The resulting values were further normalized to that of the control sample (uncut). Three independent experiments were performed for each strain.

### Analysis of Ectopic Recombination

To examine the repair kinetics of ectopic recombination, we cultured yeast cells in the pre-induction medium (YEP–Raffinose) overnight to the early log phase. Then, 2% of galactose was added to induce the HO cut that generates a single DSB on chromosome V. Samples were collected at different time points. Genomic DNA was extracted using a standard phenol extraction method and digested with *E*coRI. Purified DNA was resolved on 0.8% agarose gel followed by transfer onto a positively charged nylon membrane (Perkin Elmer). Southern blotting and hybridization with radiolabeled DNA probes were performed as described previously (Zhu et al., 2008; Chen et al., 2012). The blot was exposed in a phosphor screen. The signal on the screen was captured by scanning in an OptiQuant Cyclone Plus machine (Perkin Elmer). We quantified and normalized the pixel intensity of target bands to that of corresponding parental bands on blots. The resulting values were further normalized to that of the control sample (uncut). Three independent experiments were performed for each strain.

### Drug Sensitivity Test

Yeast cells were grown in YEPD-rich medium overnight to saturation. Undiluted cell culture and 1/10 serial dilutions of each cell culture were spotted onto YPD plates containing different DNA-damaging agents at indicated concentrations. The plates were incubated at 30°C for 3 days before analysis.

### Chromatin Immunoprecipitation

Chromatin immunoprecipitation assays were carried out as previously described (Chen et al., 2012). Cultures were grown to a density of about 1 × 10^7^ cells/ml in the pre-induction medium (YEP–raffinose), and the expression of HO endonuclease was induced by adding 2% galactose. The cells were fixed with 1% formaldehyde and incubated for 10 min at room temperature with rotation. The reaction was quenched by adding 125 mM of glycine, followed by incubating at room temperature for 5 min with rotation. The cells were lysed with glass beads in lysis buffer (50 mM HEPES, pH 7.5, 1 mM EDTA, 140 mM NaCl, 1% Triton X-100, 0.1% NaDOC, 1 mg/ml bacitracin, 1 mM benzamidine, and 1 mM PMSF) supplemented with protease inhibitors. The whole-cell extracts were sonicated with a Bioruptor (Diagenode) to shear the DNA to an average size of 0.5 kb. After centrifugation, the supernatant was collected and incubated with anti-Myc (Sigma M4439) or anti-FLAG (CST) antibody overnight at 4°C, followed by incubating with protein G-agarose beads for 3 h at 4°C. The protein-bound beads were washed twice with lysis buffer, twice with lysis buffer containing 500 mM NaCl, twice in wash buffer (10 mM Tris-HCl, pH 8.0, 1 mM EDTA, 0.25 M LiCl, 0.5% NP-40 substitute, and 0.5% NaDOC), and twice in ×1 TE. The protein–DNA complexes were eluted with elution buffer (10 mM Tris-HCl, pH 8.0, 10 mM EDTA, pH 8.0, and 1% SDS) and incubated at 65°C overnight to reverse crosslinking. The samples were digested with proteinase K at 37°C for 12 h. DNA was purified by phenol extraction and ethanol precipitation. The purified DNA samples were analyzed by real-time quantitative PCR, with primers that specifically anneal to DNA sequences located at indicated distances from the DSB, using the following conditions: 95°C for 10 min and 40 cycles of 95°C for 15 s and 60°C for 1 min.

### Western Blotting

Whole-cell yeast extracts were prepared using the trichloroacetic acid method as previously described (Chen et al., 2012). The pelleted cells from 5 ml of culture were washed once with water and resuspended in 10% trichloroacetic acid. The cells were lysed by vortexing with glass beads, and the protein lysates were pelleted by centrifugation at 12,000 g for 15 min. The pellets were washed with ice-cold 80% acetone, and proteins were dissolved in ×2 SDS sample loading buffer by boiling for 5 min. The samples were centrifuged for 5 min at 12,000 g, and the supernatant was retained as protein extract. The samples were resolved on 8% SDS-PAGE gel and transferred onto a polyvinylidene difluoride membrane (Immobilon-P; Millipore) using a semi-dry method. Anti-Myc and anti-FLAG antibodies were purchased from MBL. GAPDH was purchased from GeneTex. Anti-mouse and rabbit IgG HRP-conjugated secondary antibodies were purchased from Santa Cruz Biotechnology. Blots were developed using the Western Blotting substrate (Bio-Rad).

### MNase Digestion

MNase digestion of chromatin was performed as described (Chen et al., 2012). Yeast cells from 80 ml of culture (~2 × 10^7^ ml^−1^) were collected and washed with sterilized H_2_O. The cells were resuspended in 900 μl of sorbitol solution (1 M sorbitol, 50 mM Tris-Cl, pH 7.5), followed by the addition of 0.56 μl of β-mercaptoethanol (14.3 M) and 100 μl of zymolase 20T stock (dissolved in sorbitol solution, 25 mg/ml). The samples were incubated at 30°C with rotation for 25–40 min to digest the cell wall. Spheroplasts were collected by centrifugation at 12,000 g for 2 min and washed twice with ice-cold sorbitol solution. The pellet was resuspended with lysis buffer (0.5 mM spermidine, 1 mM β-mercaptoethanol, 0.075% Nonidet P-40, 50 mM NaCl, 10 mM Tris-HCl, pH 8.0, 5 mM MgCl_2_, 5 mM CaCl_2_, plus protease inhibitors). Then, 100 unit/ml of micrococcal nuclease (NEB, M0247) was added to each sample. After mixing, 200 μl of nuclei suspension was immediately taken out as an undigested control. The remaining samples were subjected to MNase digestion at 37°C. Aliquots of nuclei suspension were taken out at indicated time points, and the reactions were stopped by the addition of 20 mM EDTA, pH 8.0, and 1% SDS. The supernatants were collected by centrifugation at 12,000 g for 10 min, followed by Proteinase K digestion (0.1 mg/ml) at 50°C for 3 h. DNA was purified by phenol–chloroform extraction and precipitated by ethanol precipitation. DNA pellet was dissolved in ×1 TE and digested with RNase A. Equal amounts of total DNA were resolved on 1.5% agarose gels and visualized by ethidium bromide staining.

## Results

### Replication Stress Inhibits DSB End Resection, RPA Loading, and HR Repair

**Figure 1:**
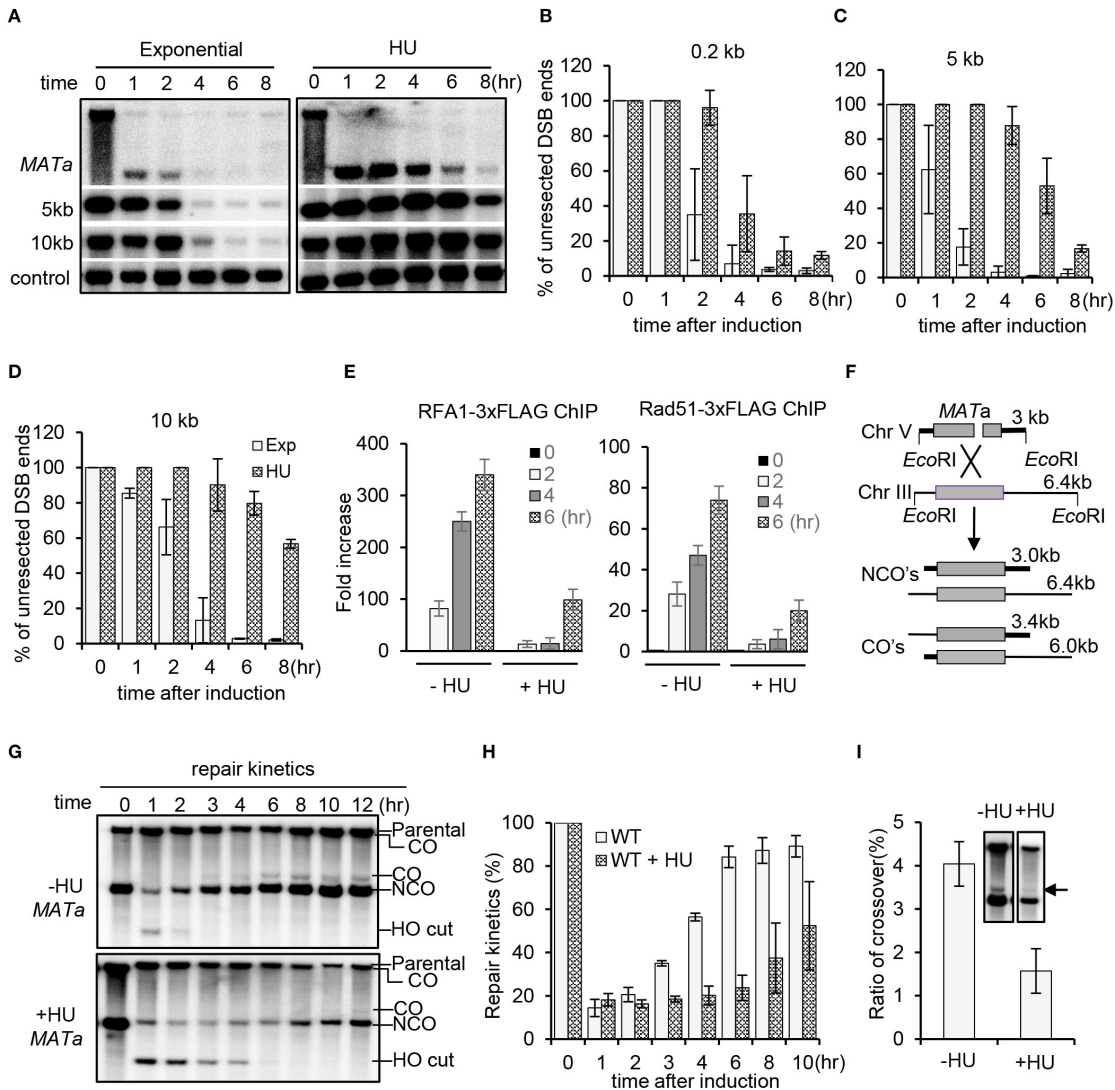
Replication stress inhibits DSB end resection and homologous recombination repair. **(A)** Southern blot analysis of resection kinetics for wild-type (WT) cells with or without hydroxyurea (HU) treatment. The location of probes is indicated. The *TRA1* probe was used as a loading control. The HO cut was induced by adding 2% galactose to early-log-phase cells grown in YP-raffinose. To induce replication stress, 200 mM HU was added into each cell culture when initiating DSB induction, and the cells were cultured under constant HU treatment. Samples were taken at indicated time points. Exp, exponential cells (without HU treatment). **(B–D)** Quantification of Southern blot presented in **(A)**. **(E)** Chromatin immunoprecipitation analysis of the recruitment of RPA−3xFLAG or Rad51–3xFLAG at DSB ends (1 kb location). DSB induction and HU treatment were carried out as described above. **(F)** Scheme showing an ectopic recombination system. CO, crossover; NCO, non-crossover. **(G,H)** Southern blot analysis and quantification of repair kinetics for WT cells with or without HU treatment. DSB induction and HU treatment were carried out as described in **(A)**. **(I)** Southern blot and quantification showing the levels of crossover products (12 h). The arrow indicates crossover products. Error bars represent the standard deviation from three independent experiments.

Previous studies have revealed that resection of 5′-ends of DSBs is inhibited upon HU treatment that causes replication stress by reducing the dNTP level (Alabert et al., 2009; Barlow and Rothstein, 2009). To verify this result, we employed a haploid yeast system wherein a single unrepairable DSB is generated at the *MAT*a locus on chromosome III upon induction of the HO endonuclease by galactose (Zhu et al., 2008). The donor sequences *HML* and *HMR* were deleted so that the cells cannot repair the DSB by HR. We compared the resection kinetics for the wild-type (WT) cells in the presence or absence of HU treatment. In unperturbed cells, the 5′-end resection proceeded normally as reported previously (Zhu et al., 2008; Chen et al., 2012). However, in the presence of constant HU treatment (200 mM), resection was completely blocked in the initial 2–4 h no matter at proximal ends or at 5 or 10 kb distal ends (Figures 1A–D). However, resection started to occur at proximal ends after 4 h and at 5 or 10 kb location after 6 h (Figures 1A–D). Therefore, the regulation of resection under constant HU treatment can be divided into two stages. In the initial 2–4 h, resection is severely suppressed, but the suppression becomes alleviated afterwards even in the presence of constant HU treatment. This is consistent with the observation that cells can bypass the S phase checkpoint and proceed through the cell cycle after prolonged HU incubation (Uzunova et al., 2014).

As a consequence, recruitment of the ssDNA binding proteins RPA and Rad51 was severely impaired within the initial 4 h, while their recruitment started to occur after 4 h (Figure 1E). Next, we evaluated DSB repair by ectopic recombination using a repair system in which a single HO-induced DSB is repaired by using the homologous sequence on chromosome III as a donor (Figure 1F) (Ira et al., 2003; Prakash et al., 2009). By Southern blot analysis, we found that the repair proceeded much slower in the presence of HU compared to that in the unperturbed cells (Figures 1G,H). Interestingly, the level of crossover products was reduced under replication stress (Figure 1I). These results together indicate that HU-induced replication stress represses resection and DSB repair by HR.

### Both Sgs1–Dna2 and Exo1 Pathways Are Repressed

**Figure 2:**
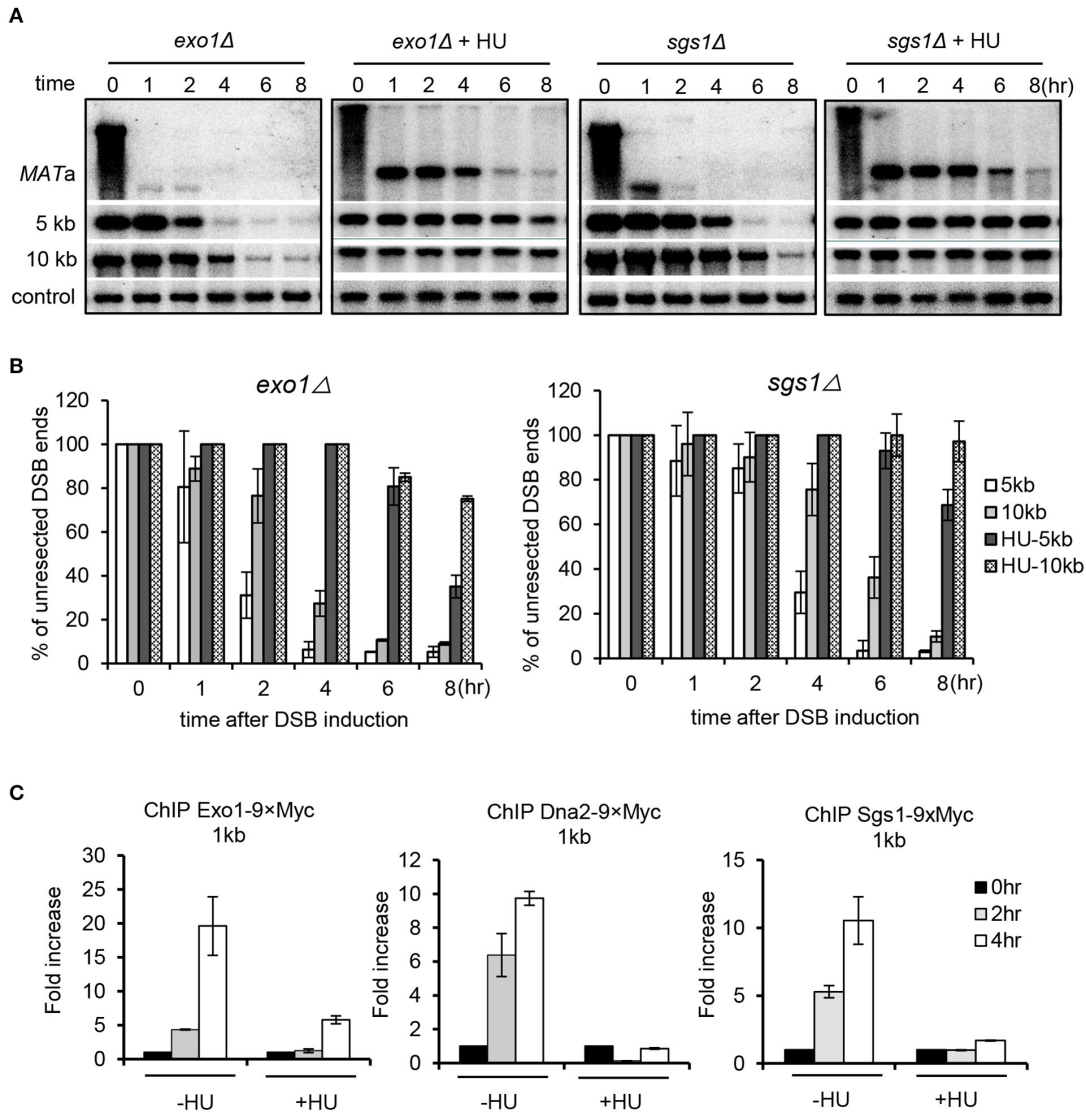
Replication stress inhibits both Sgs1/Dna2 and Exo1 resection pathways. **(A,B)** Southern blot analysis and quantification of DSB end resection for the indicated mutant cells with indicated probes. **(C)** Chromatin immunoprecipitation analysis of the recruitment of Exo1–9xMyc, Dna2–9xMyc, or Sgs1–3xFLAG at DSB ends (1 kb location). DSB induction and hydroxyurea treatment for experiments in this figure were performed as described in Figure 1A. Samples were collected at indicated time points. Error bars represent the standard deviation from three independent experiments.

Long-range resection is carried out by two partially redundant pathways mediated by Sgs1–Dna2 or Exo1 (Mimitou and Symington, 2008; Zhu et al., 2008; Cejka et al., 2010; Niu et al., 2010). To delineate which pathway was inhibited by replication stress, we compared the resection rate for *sgs1Δ* or *exo1Δ* mutant in the absence or presence of HU treatment. Compared to the unperturbed condition, replication stress severely impaired resection in the *exo1Δ* mutant, indicating that the Sgs1–Dna2 pathway was suppressed (Figures 2A,B). Similarly, resection in *sgs1Δ* cells was also severely impaired under HU treatment, indicating that the Exo1-mediated pathway was also suppressed. Consistently, recruitment of the resection enzymes Sgs1, Dna2, and Exo1 was nearly abolished at 4 h under replication stress compared to the unperturbed condition (Figure 2C). These results indicate that replication stress impairs the recruitment of the resection enzymes, thereby repressing DSB end resection.

### Mrc1 Is Critical to Mediate the Suppression of Resection Upon Replication Stress

**Figure 3:**
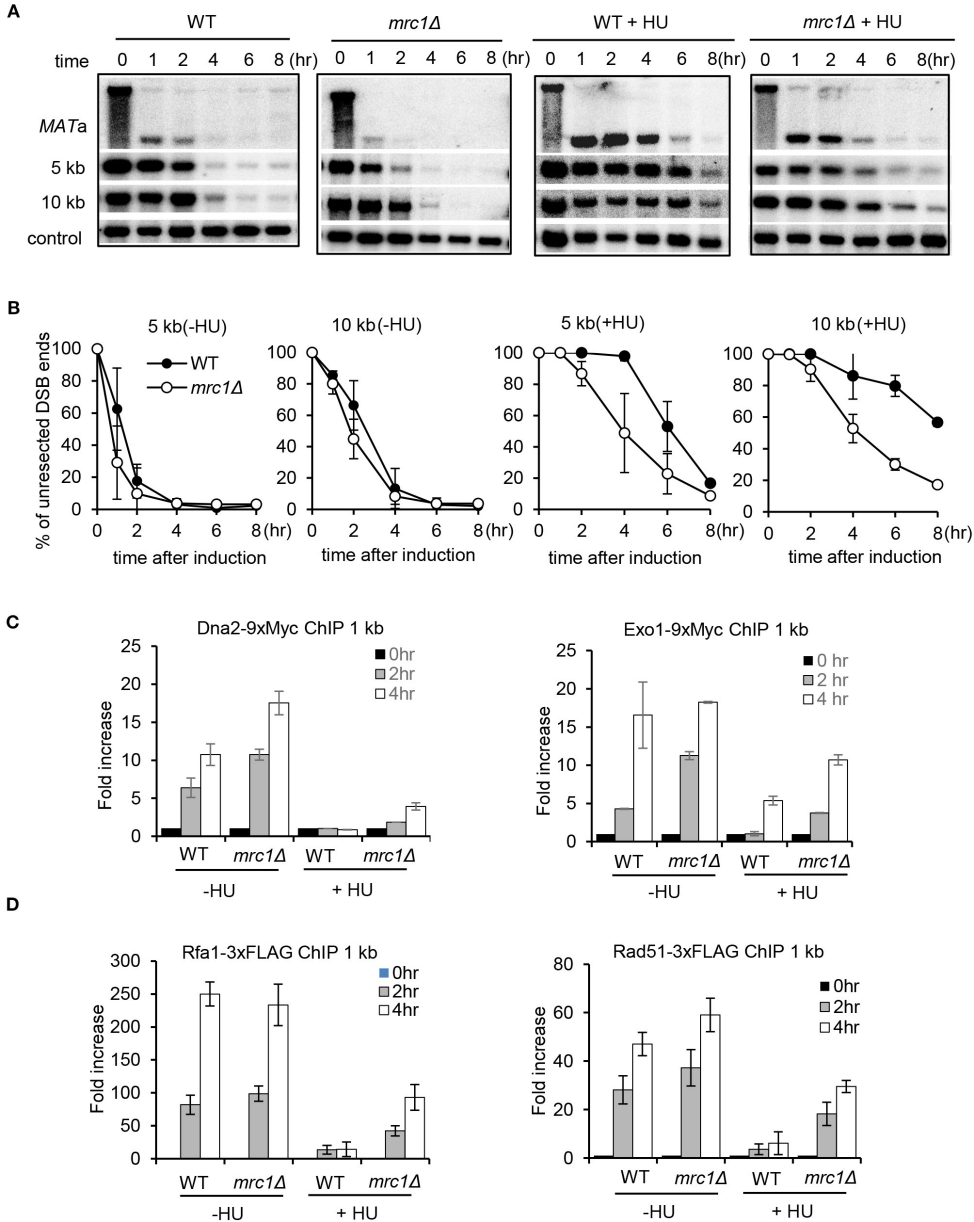
Mrc1 mediates the HU-induced inhibition on DSB end resection. **(A,B)** Southern blot analysis and quantification of DSB end resection for wild-type or *mrc1Δ* mutant cells with or without HU treatment. The location of probes is indicated. **(C,D)** Chromatin immunoprecipitation analysis of the recruitment of Dna2–9xMyc, Exo1–9xMyc, Rfa1–3xFLAG, or Rad51–3xFLAG at DSBs in indicated cells with or without HU treatment. For HU treatment in **(A–D)**, 200 mM HU was added into each sample when starting DSB induction, and the cells were cultured under constant HU treatment. Samples were collected at indicated time points after DSB induction. Error bars denote standard deviation from three independent experiments.

Mrc1 travels with the replisome and is essential to sense and activate the checkpoint upon replication stress (Tanaka and Russell, 2001; Katou et al., 2003; Smolka et al., 2006; Xu et al., 2006; Lou et al., 2008; Komata et al., 2009). We tested whether Mrc1 mediates the HU-induced repression of DSB end resection. Interestingly, we found that, in unperturbed conditions, the *mrc1Δ* mutant exhibited faster resection at proximal or distal ends when compared to WT cells (Figures 3A,B), suggesting that Mrc1 plays a role in limiting resection in cycling cells. In the presence of HU treatment, resection in *mrc1Δ* cells became slower as compared to unperturbed conditions. However, when compared to the HU-treated WT cells, resection was much faster in HU-treated *mrc1Δ* mutant cells (Figures 3A,B), indicating an important role of Mrc1 in mediating the suppression of resection. Consistently, the deletion of *MRC1* partially restored the recruitment of Dna2 and Exo1 and the loading of RPA and Rad51 4 h after HU treatment (Figures 3C,D). The levels of these proteins are comparable between WT and *mrc1Δ* cells, suggesting that the differences in their loading were not due to any changes in their protein levels (Supplementary Figure 1). Although resection occurred at late hours, the Mrc1-deficient cells still failed to repair DSBs by ectopic recombination in the presence of HU and showed hypersensitivity to HU or MMS (Supplementary Figure 2). This may reflect the role of Mrc1 in mediating the replication checkpoint and in ensuring fork progression or restart at stalled forks.

### The Replication Checkpoint Is Essential to Maintain Suppression

**Figure 4:**
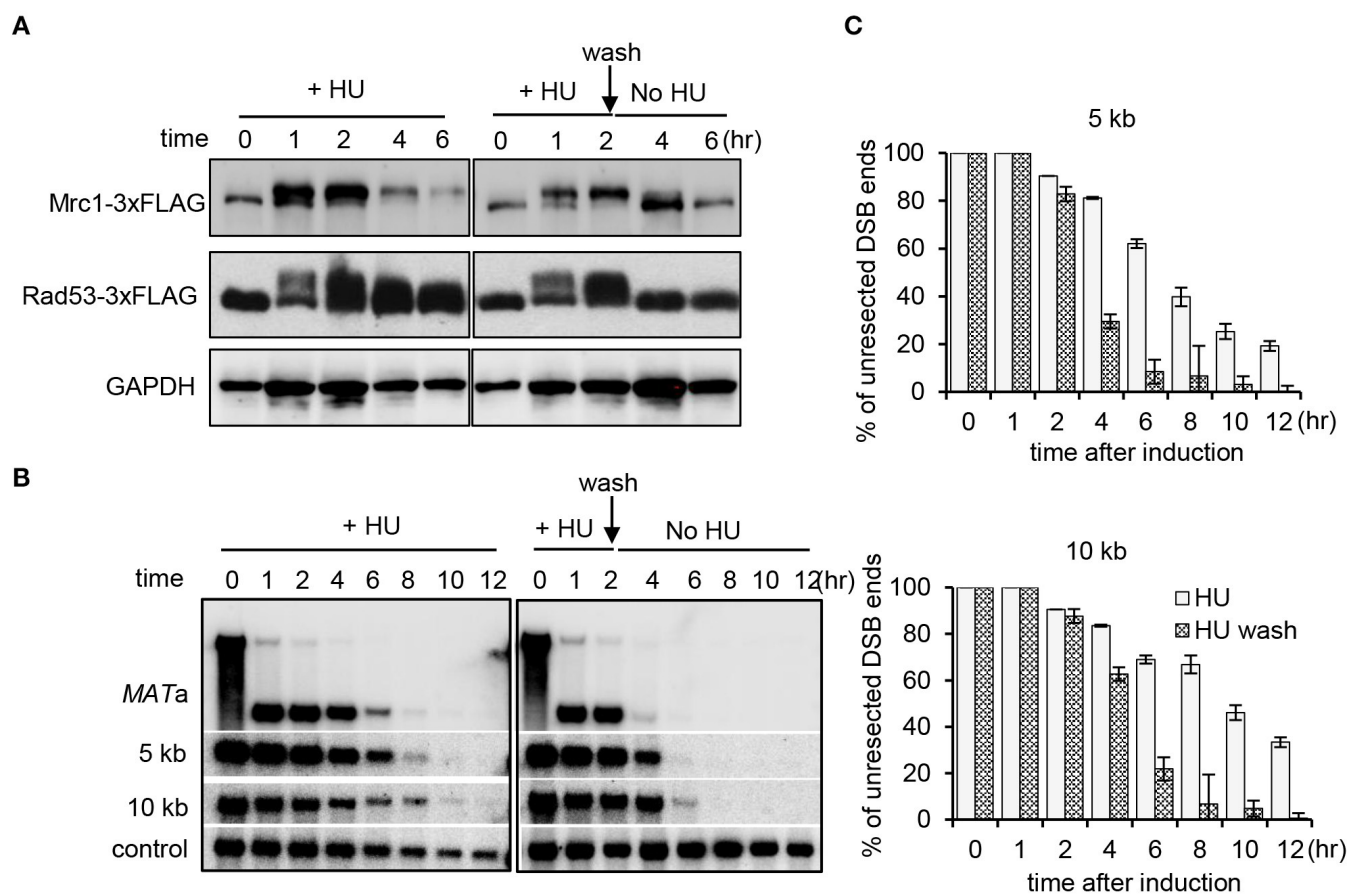
Mrc1-mediated replication checkpoint is essential for the maintenance of inhibition on resection. **(A)** Western blot showing replication checkpoint activation as indicative of the phosphorylation of Mrc1 and Rad53. GAPDH serves as a loading control. The WT cells were first treated with HU for 2 h. Then, half of the culture was washed and allowed to recover in fresh medium without HU, while the other half remained with constant HU treatment. **(B)** Southern blot analysis of double-stranded break end resection for WT cells with HU treatment or during the recovery. HU treatment was performed as described in **(A)**. Samples were taken at indicated time points. **(C)** Quantification of the Southern blot is presented in **(B)**. Error bars denote standard deviation from three independent experiments.

Next, we asked whether the Mrc1-mediated replication checkpoint is required to maintain the suppression on resection. To this end, the WT cells were first treated with HU for 2 h. Half of the culture was washed and allowed to recover in fresh medium without HU, while the other half remained with constant HU treatment. We monitored the kinetics of checkpoint activation and the progression of DSB end resection. The replication checkpoint was activated within 1 h following HU treatment as indicated by the phosphorylation of Mrc1 and Rad53 (Figure 4A). After removing HU, the checkpoint was quickly turned off since both Mrc1 and Rad53 were dephosphorylated (Figure 4A). Importantly, we observed that the removal of HU also quickly alleviated the suppression of resection (Figures 4B,C). However, under constant HU treatment, Rad53 dephosphorylation was apparently slower. Accordingly, the suppression of resection was sustained longer (Figures 4B,C). Thus, the suppression of resection correlates with the status of replication checkpoint activation. These results suggest that the Mrc1-mediated replication checkpoint signaling is essential to suppress DNA end resection and to maintain this suppression.

### Mrc1-Mediated Suppression of Resection Is Independent of Csm3, Tof1, or Ctf4

**Figure 5:**
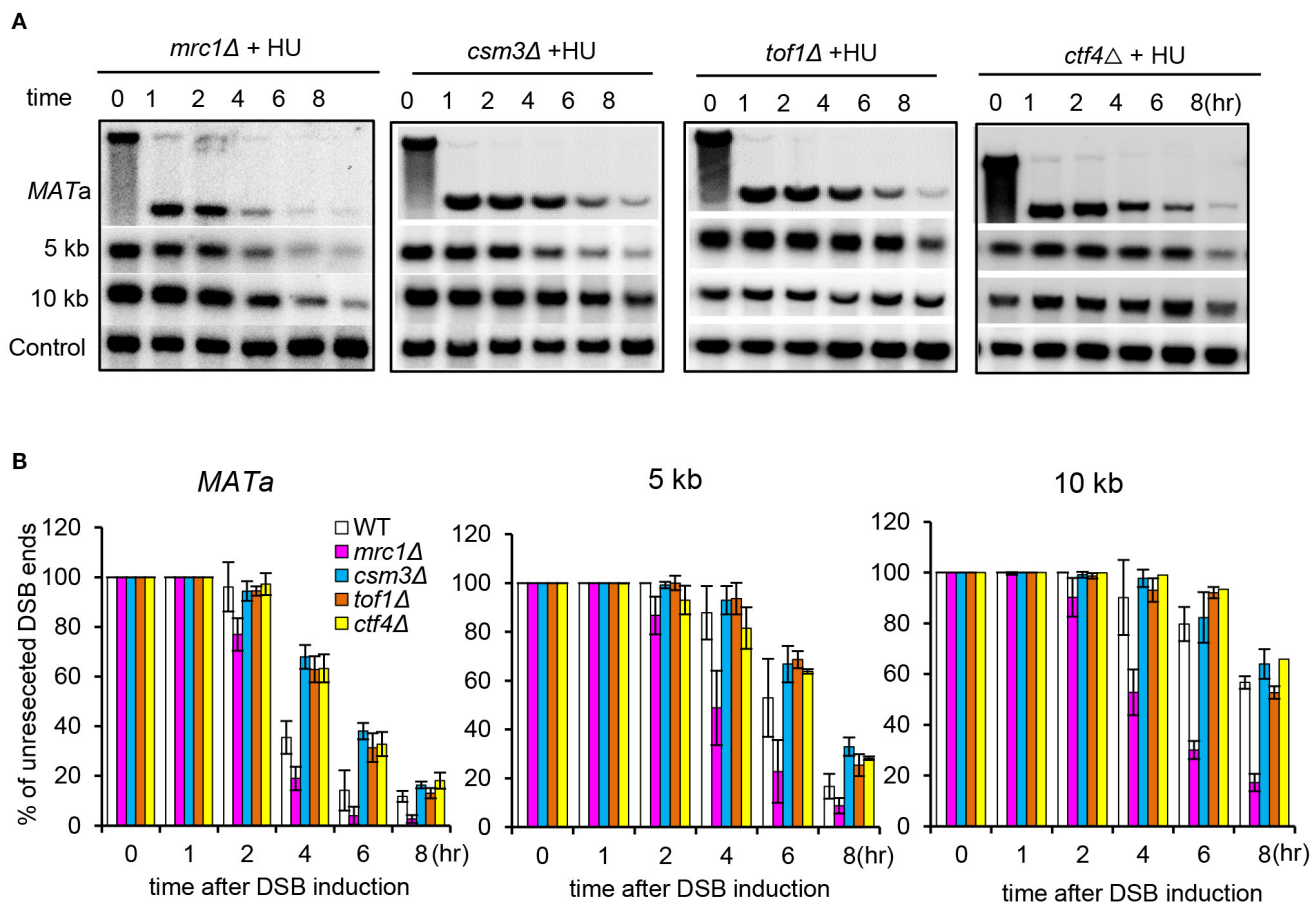
Mrc1-mediated suppression of resection is independent of Csm3, Tof1, or Ctf4. **(A,B)** Southern blot analysis and quantification of DSB end resection at indicated locations for the indicated mutant cells with HU treatment. For HU treatment, 200 mM HU was added into each sample when initiating DSB induction, and the cells were cultured under constant HU treatment. Samples were collected at indicated time points after DSB induction. Error bars denote standard deviation from three independent experiments.

The checkpoint proteins Mrc1, Csm3, and Tof1 can form a heterotrimeric complex that travels with the replication fork and mediates the activation of replication checkpoint (Katou et al., 2003; Noguchi et al., 2004; Calzada et al., 2005). We tested whether Csm3 and Tof1 are involved in the suppression of resection. We monitored resection for the *csm3Δ* or *tof1Δ* mutant cells with constant HU treatment. The result showed that the resection rate in the *csm3Δ* or *tof1Δ* mutant resembles that of WT cells, and it was significantly slower than that observed in *mrc1Δ* cells (Figures 5A,B). It was recently reported that the replisome component Ctf4 functions as a key regulator suppressing DSB end resection at arrested forks in rDNA region (Sasaki and Kobayashi, 2017). We examined whether it affects DSB end resection under replication stress. We found that deletion of *CTF4* did not alleviate the inhibition on resection (Figures 5A,B). These results together suggest that the Mrc1-mediated suppression of resection under replication stress is largely independent of Csm3, tof1, or Ctf4.

### The Deletion of *RAD9* Fails to Restore Resection Under Replication Stress

**Figure 6:**
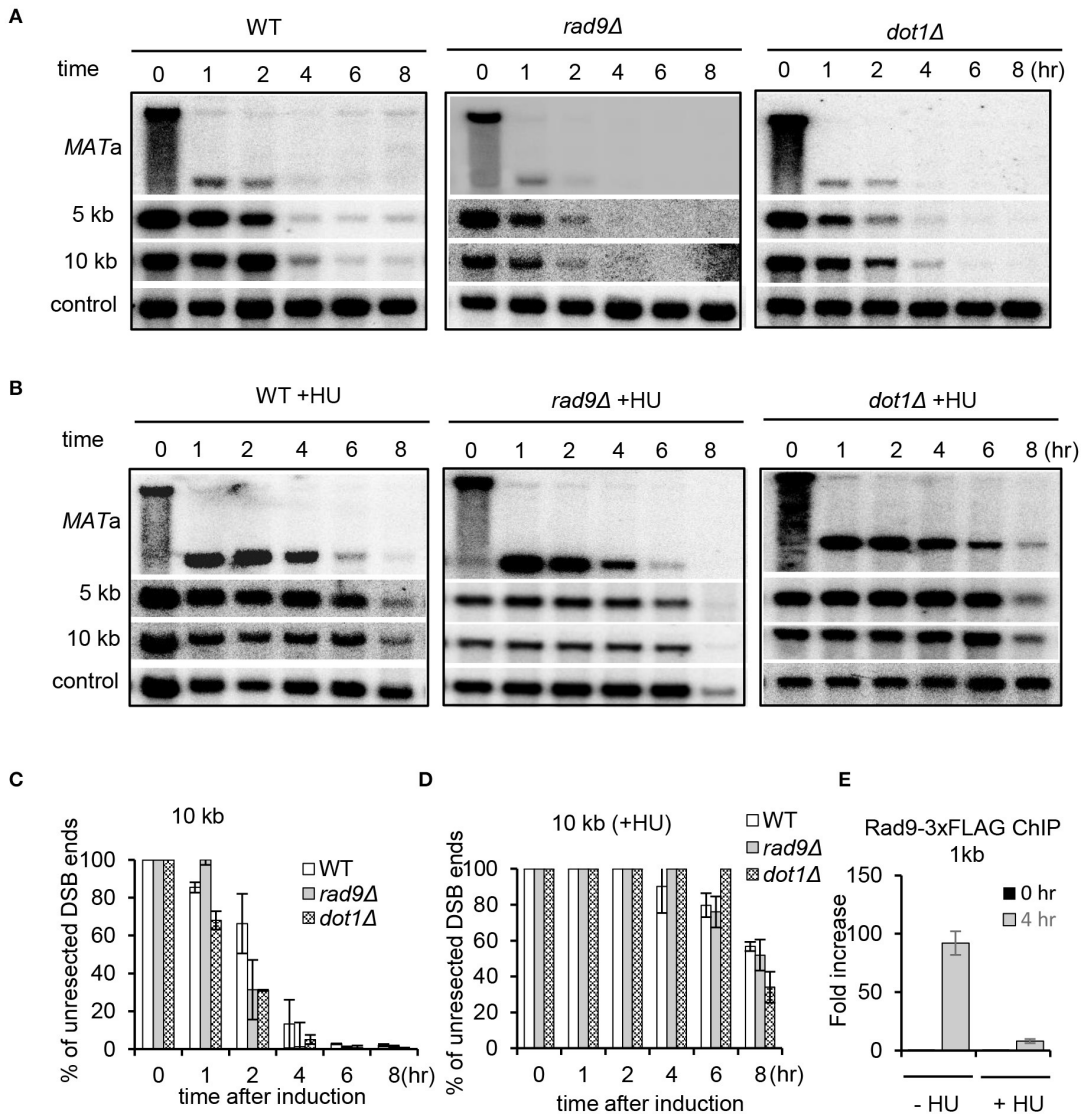
The deletion of *RAD9* fails to increase resection under replication stress. **(A,B)** Southern blot analysis and quantification of DSB end resection for the indicated mutant cells with or without HU treatment. HU treatment was performed as described in Figure 1A. **(C,D)** Quantification of the Southern blot shown in **(A)** and **(B)**. **(E)** Chromatin immunoprecipitation analysis of Rad9–3xFLAG recruitment at DSB ends (1 kb) in the absence or presence of HU treatment. Error bars denote standard deviation from three independent experiments.

Rad9, the mediator protein for the DNA damage checkpoint, is known to form a physical barrier suppressing resection at DSB ends (Lazzaro et al., 2008; Chen et al., 2012). Rad9 is recruited to damaged chromatin *via* associating with Dpb11, histone H3 methylated on K79, or H2A phosphorylated on Serine 129 (Wysocki et al., 2005; Toh et al., 2006; Grenon et al., 2007; Puddu et al., 2008; Pfander and Diffley, 2011). Consistent with previous studies, we found that lack of either Rad9 or Dot1, the methyltransferase for H3K79 methylation, resulted in faster resection as compared to WT cells in unperturbed conditions (Figures 6A,C) (Lazzaro et al., 2008; Chen et al., 2012). We then tested whether deletion of *RAD9* or *DOT1* could bypass the suppression on resection under replication stress. However, we found that resection in both *rad9Δ* and *dot1Δ* mutant cells remained inhibited in the presence of HU as seen in WT cells (Figures 6B,D). Thus, the replication stress-induced suppression of resection cannot be bypassed by removing H3K79 methylation or Rad9. However, we noted that Rad9 was not recruited properly at DSBs under replication stress as compared to that in unperturbed conditions (Figure 6E). These results together reveal a unique role of Mrc1-mediated replication checkpoint in inhibiting DSB end resection.

### Replication Stress Induces Global Chromatin Compaction and Impairs the Recruitment of Chromatin Remodeling Factors

**Figure 7:**
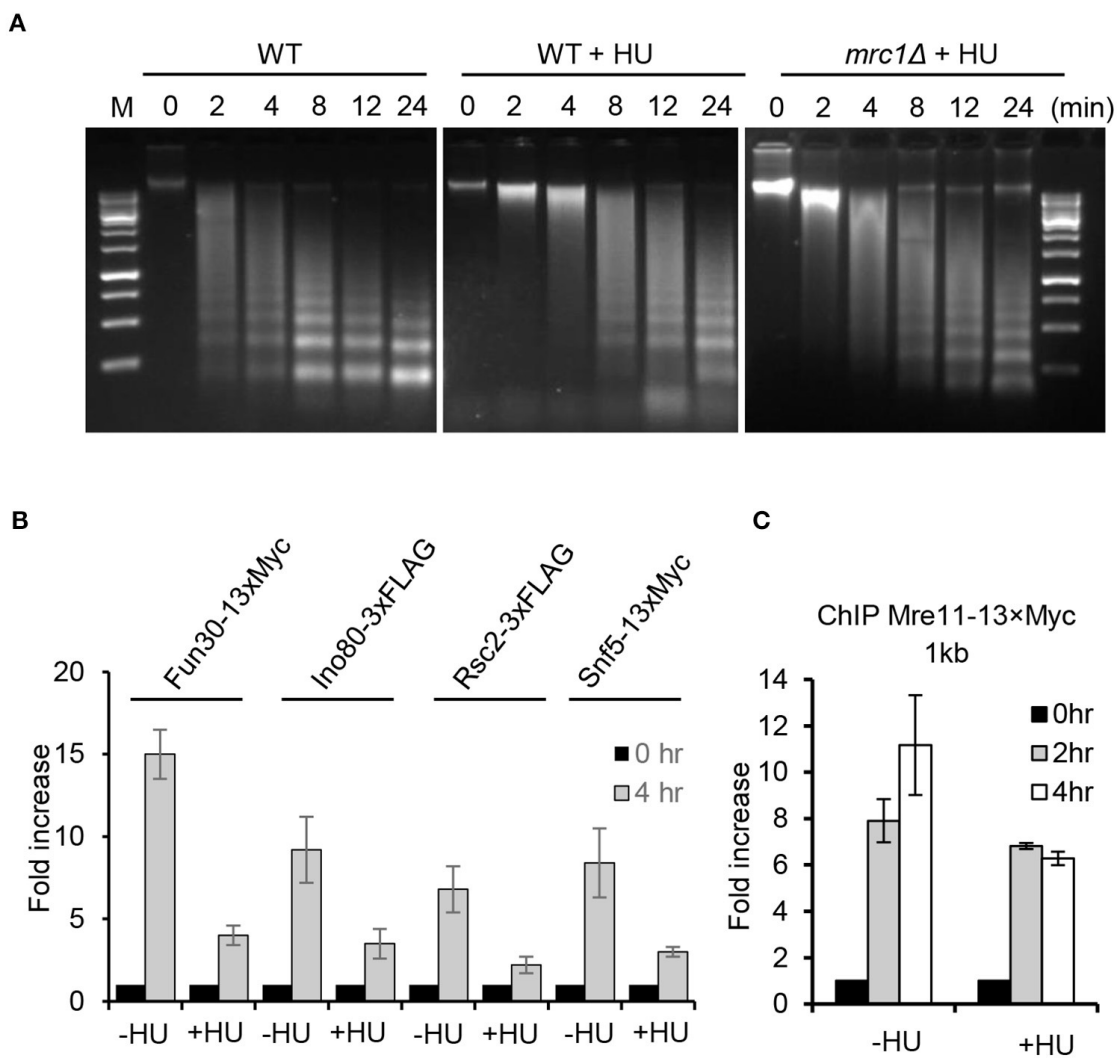
Replication stress induces global chromatin compaction. **(A)** MNase digestion of chromatin DNA for the indicated cells with or without HU treatment. For HU treatment, the cells were treated with 200 mM HU for 2 h. Chromatin samples were collected after different time points of MNase digestion. **(B,C)** Chromatin immunoprecipitation analysis of Fun30–13xMyc, Ino80–3xFLAG, Rsc2–3xFLAG, Snf5–13xMyc, or Mre11–13xMyc recruitment at double-stranded break ends (1 kb) in the absence or presence of HU treatment. For HU treatment, the cells were cultured in the presence of 200 mM HU for 2 or 4 h. Samples were taken at indicated time points. Error bars denote standard deviation from three independent experiments.

In fission yeast, it was recently reported that replication stress induces the deacetylation of H2B-K33Ac and tri-methylation on H3K79, thereby triggering chromatin compaction (Feng et al., 2019). We compared the chromatin structure of WT cells in the presence or absence of HU treatment using the MNase digestion. We observed that, compared to untreated cells, HU treatment (2 h) led to the apparent compaction of chromatin as reflected by the poor digestion of chromatin DNA by MNase (Figure 7A). Importantly, we noted that HU treatment also resulted in chromatin compaction in the *mrc1Δ* mutant, but it was less severe than that in WT cells, suggesting that Mrc1 is required to induce condensed chromatin under replication stress.

Several ATP-dependent chromatin remodelers are recruited to DSBs, where they act to remodel local chromatin structure and to promote DSB repair (Chai et al., 2005; Shim et al., 2007; Chen et al., 2012; Costelloe et al., 2012; Seeber et al., 2013; Wiest et al., 2017). Consistently, we observed enrichment of the chromatin remodelers Ino80, RSC, Fun30, or Snf5 at DSB ends 4 h following break induction in the absence of HU (Figure 7B). However, their enrichment was significantly reduced in cells with constant HU treatment. Next, we tested whether the loading of Mre11, which recognizes DNA breaks, was affected under replication stress. We noted that Mre11–3xFLAG was efficiently recruited to DSBs in unperturbed cells, while its recruitment was slightly impaired in HU-treated cells at 4 h after DSB induction, suggesting that recognition of DSBs by Mre11 on the compacted chromatin was modestly affected (Figure 7C). These results together suggest that the Mrc1-mediated replication checkpoint induces global chromatin condensation that limits the access of chromatin remodelers, resection machinery, and repair proteins to the damaged site, leading to the suppression of HR repair.

## Discussion

Cells in the S phase are particularly vulnerable to genotoxic stresses that can lead to replication stress and genome instability, which are hallmarks of cancer. It is known that HR proteins are important to protect and restart stressed forks (Prado, 2014; Branzei and Szakal, 2017; Pardo et al., 2017). Paradoxically, the Mrc1-dependent checkpoint was shown to prevent HR repair at DSBs or arrested forks (Prado, 2014; Garcia-Rodriguez et al., 2018). How the replication checkpoint inhibits HR remains unclear. In this study, we showed that the replication stress-induced checkpoint mediated by Mrc1 represses both Sgs1/Dna2 and Exo1 resection pathways, leading to defective RPA and Rad51 loading and HR repair. Mechanistically, we found that the Mrc1-dependent checkpoint induces global compaction of chromatin that blocks the access of multiple chromatin remodeling factors and resection machinery. We also show that the suppression of resection in the S phase is specifically mediated by the replication checkpoint mediator Mrc1 but is independent of the replisome components Csm3, Tof1, or Ctf4 or the DNA damage checkpoint mediator Rad9.

Why do cells inhibit HR repair, given that HR proteins are required to protect or restart stressed forks? It was proposed that the replication checkpoint may differentially regulate recombination at DSBs and replication forks (Alabert et al., 2009). Cells must prevent undesired or premature HR repair that can lead to errors, loss of heterozygosity, or genome arrangement, especially when it is initiated with templates other than the sister chromatid (Alcasabas et al., 2001; Branzei and Szakal, 2017). The suppression of HR provides a time window of opportunity to protect and stabilize the forks, allowing repair or bypass of the DNA lesion before fork restart (Barlow and Rothstein, 2009). The spatial–temporal separation of HR at DSBs and stressed forks allow cells to repair the damaged DNA after completing the S phase, whereupon the stress is relieved (Pardo et al., 2017).

We provided evidence that the replication stress-induced suppression of resection depends on Mrc1, but not Rad9. Consistently, we found that Rad9 is not recruited to DSBs under HU treatment (Figure 6E). These two mediators play distinct functions in sensing the checkpoint in the S phase. The Mrc1-dependent pathway primarily monitors the state of the replisome and is considered to be associated closely with replication forks (Garcia-Rodriguez et al., 2018). This pathway is independent of the exonuclease Exo1 that is involved in expanding ssDNA gaps (Garcia-Rodriguez et al., 2018). In contrast, more ssDNA is accumulated behind the fork upon MMS or UV exposure, which activates the Rad9-dependent pathway. This pathway senses DNA lesions in the daughter-strand gap left behind the replisome and in an Exo1-dependent manner (Garcia-Rodriguez et al., 2018).

Mrc1 forms a complex with Csm3 and Tof1, and the association of Mrc1 with replication forks partially depends on Csm3 and Tof1 (Tourriere et al., 2005; Bando et al., 2009; Prado, 2014; Pardo et al., 2017). This complex is critical for proper activation of the replication checkpoint and protection or restart of stalled forks (Tourriere et al., 2005; Bando et al., 2009; Prado, 2014; Pardo et al., 2017). However, there are multiple pieces of evidence showing that these proteins also have non-overlapping functions. For example, Mrc1-deficient cells are more sensitive to HU than *tof1Δ* mutant cells (Tourriere et al., 2005). Compared to Tof1, Mrc1 is more important in preventing the fragility and instability of long CAG repeats (Gellon et al., 2019). Notably, it was reported that Mrc1 plays a major role in the activation of the replication checkpoint, while Tof1 likely only plays an indirect role in this process (Tourriere et al., 2005). These differences may explain why the replication stress-induced suppression of resection depends on Mrc1, but not Csm3, Tof1, or Ctf4.

Notably, we observed that HU treatment induces global chromatin compaction that partially depends on Mrc1, leading to the blockage of the access of chromatin remodeling factors and resection machinery (Figures 7A,B). This is consistent with the observation that replication stress also triggers chromatin compaction in fission yeast (Feng et al., 2019). It was reported that replication stress induces the deacetylation of histone H2B-K33ac by Clr6 and the enrichment of H3K9 tri-methylation at stalled forks, which contributes to the formation of a compacted chromatin environment (Feng et al., 2019). Constitutive mimic acetylation of H2B-K33ac leads to uncoupling of replicative helicase and DNA polymerases and replication fork instability (Feng et al., 2019). Thus, replication stress-triggered chromatin compaction is likely a conserved cellular response. However, whether Mrc1-dependent chromatin compaction in yeast is affected by similar histone modifications remains to be determined.

Chromatin compaction can also result from other mechanisms. For example, in mammalian cells, DSBs can induce temporary chromatin compaction that involves the macro-histone variant macroH2A1 and demethylation of H3-K9 (Khurana et al., 2014; Oberdoerffer, 2015). This change facilitates the accumulation of BRCA1 at DSBs (Khurana et al., 2014; Oberdoerffer, 2015). In yeast mitosis, chromatin compaction can be defined as two mechanistically distinct processes (Kruitwagen et al., 2015): one is called chromatin compaction, mediated by Ipl1-dependent H3S10 phosphorylation that recruits the deacetylase Hst2 to remove H4K16 acetylation, leading to a tighter packing of neighboring nucleosomes (Wilkins et al., 2014; Kruitwagen et al., 2015); the other is a condensin-dependent axial chromosome contraction process that promotes the long-range contraction of chromosomes (Kruitwagen et al., 2015; Hirano, 2016). It was also reported that lack of HMO1, the mobile chromatin-binding protein, confers chromatin hypersensitivity to nuclease and creates a more accessible chromatin state (Panday and Grove, 2017). Therefore, it is important to determine whether the replication stress-induced chromatin compaction in yeast is related to these factors.

## Data Availability Statement

The original contributions presented in the study are included in the article/Supplementary Material, further inquiries can be directed to the corresponding author/s.

## Author Contributions

PX designed and conducted the majority of the experiments. YD, JZ, ZZ, ZL, YW, and ML constructed some yeast strains and participated in DNA extraction. XC supervised the study. PX and XC wrote the manuscript. XZ discussed the data and critically read the manuscript. All authors contributed to the article and approved the submitted version.

## Conflict of Interest

The authors declare that the research was conducted in the absence of any commercial or financial relationships that could be construed as a potential conflict of interest.
