# Supplementary material for: Mrc1-Dependent Chromatin Compaction Represses DNA Double-Stranded Break Repair by Homologous Recombination Upon Replication Stress
Source: Front Cell Dev Biol. 2021 Feb 15;9:630777. doi: 10.3389/fcell.2021.630777 (PMC7928320; doi:10.3389/fcell.2021.630777)
Supplement: Supplementary file 1 [file Table_1.DOCX]

| Strain  name | Parental  strain | Genotype | Source |
| --- | --- | --- | --- |
| JKM139 |  | *MATa ho hml::ADE1 hmr::ADE1 ade1-100 leu2-3,112 trp1::hisG lys5 ura3-52 ade3::GAL::HO* | 1 |
| yCW007 | JKM139 | *RFA1-3xFLAG-KanMX* | This study |
| yCW005 | JKM139 | *RAD51-3xFLAG-KanMX* | This study |
| yXC219 | JKM139 | *exo1::KanMX* | This study |
| yXC458 | JKM139 | *sgs1::NatMX* | 2 |
| yZZ042 | JKM139 | *EXO1-9xMYC-TRP1* | 3 |
| yXC002 | JKM139 | *DNA2-9xMYC-TRP1* | 2 |
| yZL015 | JKM139 | *SGS1-3xFLAG-KanMX* | This study |
| yPX001 | JKM139 | *mrc1::KanMX* | This study |
| yPX002 | JKM139 | *mrc1::KanMX DNA2-9xMYC-TRP1* | This study |
| yPX003 | JKM139 | *mrc1::KanMX EXO1-9xMYC-TRP1* | This study |
| yPX006 | JKM139 | *mrc1::TRP1 RFA1-3xFLAG-KanMX* | This study |
| yPX005 | JKM139 | *mrc1::TRP1 RAD51-3xFLAG-KanMX* | This study |
| yPX018 | JKM139 | *MRC1-3xFLAG-KanMX* | This study |
| yCW004 | JKM139 | *RAD53-3xFLAG-KanMX* | This study |
| yPX050 | JKM139 | *csm3::KanMX* | This study |
| yPX051 | JKM139 | *tof1::KanMX* | This study |
| yPX070 | JKM139 | *ctf4::KanMX* | This study |
| yXC108 | JKM139 | *rad9::NatMX* | 2 |
| yXC780 | JKM139 | *dot1::KanMX* | 2 |
| yXC607 | JKM139 | *FUN30-13xMYC-KanMX* | 2 |
| yCW041 | JKM139 | *INO80-3xFLAG-KanMX* | 2 |
| yCW020 | JKM139 | *RSC2-3xFLAG-KanMX* | This study |
| yPX072 | JKM139 | *SNF5-13xMYC-KanMX* | This study |
| yCW017 | JKM139 | *MRE11-13xMYC-KanMX* | This study |
| tGI354 |  | *MATa-inc arg5,6::MATa-HPH ade3::GAL::HO hmr::ADE1 hml::ADE1 ura3-52* | 4 |
| yPX008 | tGI354 | *mrc1::TRP1* | This study |

Supplementary Table 1. Yeast strains.

1. Lee, S. E. et al., Saccharomyces Ku70, mre11/rad50 and RPA proteins regulate adaptation to G2/M arrest after DNA damage.(1998). Cell 94 (3): 399.

# 2. Chen. X. et al., The Fun30 ATP-dependent nucleosome remodeler promotes resection of DNA double-strand break ends. Nature, 2012, 489(7417): 576-580

3. Zhu, Z. et al., Sgs1 helicase and two nucleases dna2 and exo1 resect DNA doublestrand break ends.(2008) Cell 134 (6): 981

4. Ira G. et al., Srs2 and Sgs1-Top3 suppress crossovers during double-strand break repair in yeast. (2003) [Cell.](https://www.ncbi.nlm.nih.gov/pubmed/?term=ira+2003+srs2)115(4):401-11.
