# Supplementary figures and images for: Mrc1-Dependent Chromatin Compaction Represses DNA Double-Stranded Break Repair by Homologous Recombination Upon Replication Stress
Source: Front Cell Dev Biol. 2021 Feb 15;9:630777. doi: 10.3389/fcell.2021.630777 (PMC7928320; doi:10.3389/fcell.2021.630777)

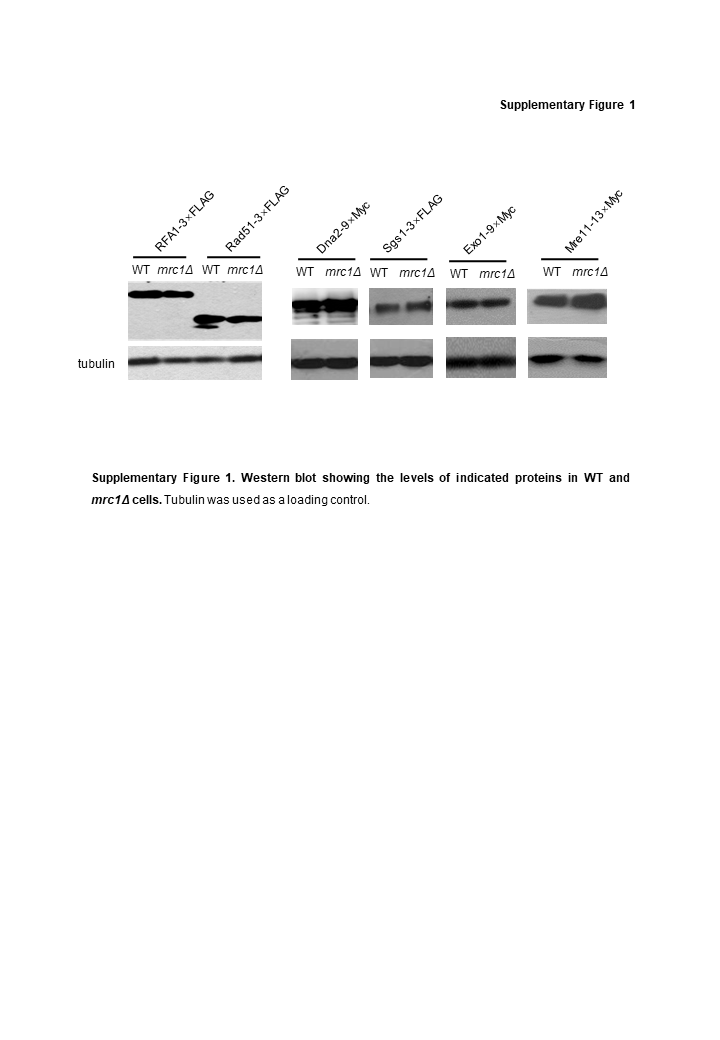

Supplement: Supplementary file 2 [file Image_1.TIF]

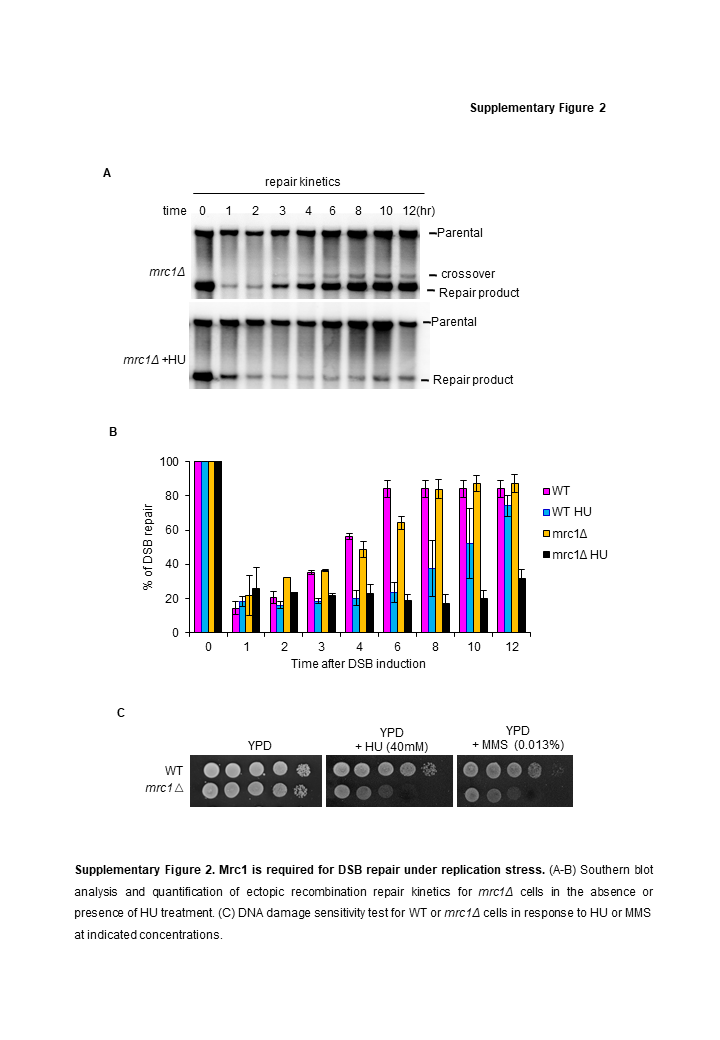

Supplement: Supplementary file 3 [file Image_2.TIF]
